# Georges Guillain (1876–1961)

**DOI:** 10.1007/s00415-016-8226-9

**Published:** 2016-07-06

**Authors:** Krzysztof Pietrzak, Andrzej Grzybowski, Jacek Kaczmarczyk

**Affiliations:** 1 Department of Orthopaedics and Traumatology, University of Medical Sciences, Poznan, Poland; 2 Department of Ophthalmology, Poznań City Hospital, Poznan, Poland; 3 Department of Ophthalmology, University of Warmia and Mazury, Olsztyn, Poland

**Fig. 1:**
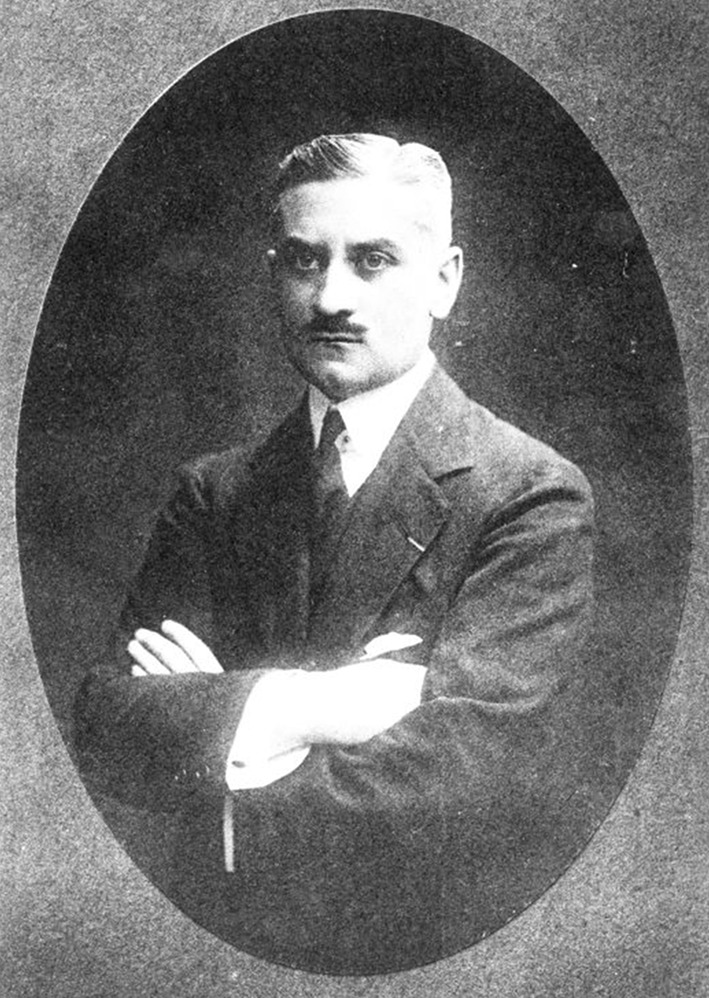
Georges Guillain (1876–1961). Permission: BIU Santé, Paris

Georges Guillain (Fig. 1) was born on 3 March, 1876 in Rouen in France. His father, Louis Guillain, was an engineer, and his mother, Gabrielle Guillain, was a daughter of a wealthy industrialist. After completing general education, Guillain started to study medicine in his hometown. After two years, in 1895, he moved to Paris to continue his medical studies.

During his clinical classes in Paris hospitals, he became more and more interested in neurology, which became the topic of his first research paper published in 1898 [1] on damage to the plexus brachialis. The person who at that time had the greatest impact on Guillain, as a developing scientist was Pierre Marie (1853–1940).

In 1902, Guillain received his Ph.D. for a thesis on syringomyelia [2]. In the same year, he went on a research trip to the United States, which was a highly unusual decision, as at that time, it was Paris that was considered to be the world’s neurology centre. This trip left Guillain very impressed by philanthropists’ substantial contributions to the advancement of science.

From 1903, Guillain worked in a number of neurology departments. In 1906, he was awarded a doctor’s diploma, which was a qualification necessary for holding an independent position in a hospital. Afterwards, Guillain worked in various hospitals in Paris, including Salpêtrière. This was the place, where he became head of the neurology department in 1910, following *concours d’agrégation,* a stage in career advancement in science, similar to professorship. This was possible due to the overwhelming support of medical students.

In 1916, following the outbreak of World War I, Guillain became head of the Neurology Centre of the sixth French Army, where he met Jean Alexander Barré (1880–1967). The meeting marked the beginning not only of long-lasting research cooperation but also of friendship. In 1916, within only 2 weeks, they admitted to an army hospital with two soldiers presenting with paraesthesias, progressive fatigue, and walking disorders [3]. The symptoms developed into a neurological disorder, affecting the limbs and the face, including such problems as the weakening of muscle function and tone, weaker neurological reflexes, symptoms of hyperalgesia, and, finally, progressive weakening of limb muscle strength. Clinical observations were corroborated by electrophysiological studies done by a psychiatrist, André Strohl (1887–1977). The tests demonstrated weakened neurological reflexes as well as weakened and delayed muscle reflexes. However, the medical knowledge of that time did not allow the interpretation that such symptoms resulted from peripheral nerve demyelination. The researchers also examined the cerebrospinal fluid and found elevated protein levels but normal cellular composition, which helped them to exclude meningitis. Guillain, Barré, and Strohl believed that the disorders were caused by damage to nerve roots, nerves, and muscles either of infectious or of toxic origin. With the passage of time, the first patient fully recovered, while the other patient’s symptoms significantly subsided. The doctors believed that the cases they described were different from the cases described earlier by Octave Landry (1826–1865) [4]. Landry’s cases showed a significant heterogeneity, their course was much more serious, the outcome was poor, and in several cases, patients died.

Guillain and Barré’s experience from the war period resulted in studies on damage to the spinal column and the spine, and accompanying neurological symptoms. Guillain and Barré also developed a set of indications for surgery which were based on neurological symptoms [5, 6]. In addition, Guillain also challenged the view that hysteria was an underlying cause of many neurological symptoms, as at that time, hysteria was a ‘fashionable’ condition, all too often overused in diagnosing patients [5]. After the war, Guillain resumed his work as a neurologist at the Charité hospital. In 1923, he was reinstated as professor at the Salpêtrière neurology department.

The name “Guillain–Barré syndrome” was first used during a 1927 neurology congress. The congress presentation was preceded by a speech by Barré, in which he omitted Strohl’s name. Similarly, Strohl was not mentioned as a co-author of the first publication from 1916, which lends support to the claim that Barré’s omission was a deliberate one. In this way, the name “Guillain–Barré syndrome” gained ground. However, if the name of the syndrome was to recognize objectively merits of all the researchers who contributed to its description, the condition should be named Guillain–Barré–Strohl–Landry syndrome.

Guillain’s contribution to medicine is also evident in the number of eponyms derived from his name. He described a symptom typical of meningitis [7], a symptom present in syphilis of the central nervous system (Guillain–Thaone syndrome), and described an anatomical structure in the cerebellum (Guillain–Mollaret triangle) [8], as well as one of choreiform symptoms (Guillain–Bertrand–Lereboullet syndrome) [9]. He also discovered a symptom associated with tumours of the nasopharynx and the base of the skull (Guillain–Alajouanine–Garcin syndrome) [10]. Finally, he described one of diagnostic reactions in laboratory tests of cerebrospinal fluid (Guillain–Laroche–Lechelle reaction).

Guillain continued to work in Salpêtrière until his retirement in 1947. He was one of the best known and influential neurologists of his time. A member of a number of French and foreign scientific societies, Guillain was also awarded the Legion of Honour in 1947. In 1909, Guillain married Juliette Chauffard (1885–1941). The couple had five daughters. Georges Guillain died in Paris on 29 June 1961.
